# Design of β-Keto Esters with Antibacterial Activity: Synthesis, In Vitro Evaluation, and Theoretical Assessment of Their Reactivity and Quorum-Sensing Inhibition Capacity

**DOI:** 10.3390/ph16101339

**Published:** 2023-09-22

**Authors:** Maximiliano Martínez-Cifuentes, Emmanuel Soto-Tapia, Camila Linares-Pipón, Ben Bradshaw, Paulina Valenzuela-Hormazabal, David Ramírez, Patricio Muñoz-Torres, Claudio Parra

**Affiliations:** 1 Departamento de Química Orgánica, Facultad de Ciencias Químicas, Universidad de Concepción, Edmundo Larenas 129, Concepción 4070371, Chile; emsoto2019@udec.cl (E.S.-T.); clinares@udec.cl (C.L.-P.); 2 Laboratori de Química Orgánica, Facultat de Farmàcia, IBUB, Universitat de Barcelona, Av. Joan XXIII, s/n, 08028 Barcelona, Spain; benbradshaw@ub.edu; 3 Departamento de Farmacología, Facultad de Ciencias Biológicas, Universidad de Concepción, Concepción 4030000, Chile; paulinvalenzuela@udec.cl (P.V.-H.); dramirezs@udec.cl (D.R.); 4 Laboratorio de Patología Vegetal y Bioproductos, Facultad de Ciencias Agronómicas, Universidad de Tarapacá, Av. General Velásquez 1775, Arica 1000000, Chile

**Keywords:** β-keto esters, DFT, docking, LasR and LuxS, quorum sensing

## Abstract

This work proposes the design of β-keto esters as antibacterial compounds. The design was based on the structure of the autoinducer of bacterial quorum sensing, *N*-(3-oxo-hexanoyl)-l-homoserine lactone (3-oxo-C6-HSL). Eight β-keto ester analogues were synthesised with good yields and were spectroscopically characterised, showing that the compounds were only present in their β-keto ester tautomer form. We carried out a computational analysis of the reactivity and ADME (absorption, distribution, metabolism, and excretion) properties of the compounds as well as molecular docking and molecular dynamics calculations with the LasR and LuxS quorum-sensing (QS) proteins, which are involved in bacterial resistance to antibiotics. The results show that all the compounds exhibit reliable ADME properties and that only compound **7** can present electrophile toxicity. The theoretical reactivity study shows that compounds **6** and **8** present a differential local reactivity regarding the rest of the series. Compound **8** presents the most promising potential in terms of its ability to interact with the LasR and LuxS QS proteins efficiently according to its molecular docking and molecular dynamics calculations. An initial in vitro antimicrobial screening was performed against the human pathogenic bacteria *Pseudomonas aeruginosa* and *Staphylococcus aureus* as well as the phytopathogenic bacteria *Pseudomonas syringae* and *Agrobacterium tumefaciens*. Compounds **6** and **8** exhibit the most promising results in the in vitro antimicrobial screening against the panel of bacteria studied.

## 1. Introduction

In the last few decades, the emergence of antibiotic-resistant bacteria has become a significant concern for developing economies and global health. The World Health Organization (WHO) has published reports showing an increasing worry about this problem, which is predicted to worsen in the coming years [1]. The primary cause behind this development is the excessive use of antibiotics in both human and animal treatment and their widespread application in the commercial industry [2,3]. Although many countries have implemented strict regulations on antibiotic consumption, these measures have yet to halt antibiotic resistance’s continuous development effectively. As a result, cases of multidrug-resistant strains of common bacteria, such as *Escherichia coli*, *Streptococcus pneumoniae*, and nontyphoidal *Salmonella*, have been documented worldwide [1]. It is essential to address this issue through research, developing new and more robust therapeutic strategies and implementing methods for bacterial control in industry and agriculture. This will ensure access to food and health resources for future generations [1,3].

One promising avenue of research is interfering with bacterial cell communication, known as quorum sensing (QS), which synchronises the behaviours of the individual cells within a multicellular community [4,5]. This process relies on producing, releasing, and detecting small diffusible signalling molecules called autoinducers. In Gram-negative bacteria, QS is regulated by a two-component system, including synthesizing the autoinducer with LuxI homologs and the autoinducer-dependent transcriptional activator, a LuxR homolog [6]. Many pathogenic bacteria use QS to facilitate pathogenesis, producing the virulence factors necessary for host infection or evading the immune system by coordinating swarming behaviour and forming biofilms [7,8]. Therefore, interfering with bacterial communication has become an attractive target for developing new therapies in medicine and the agricultural and aquacultural industries.

**Figure 1:**
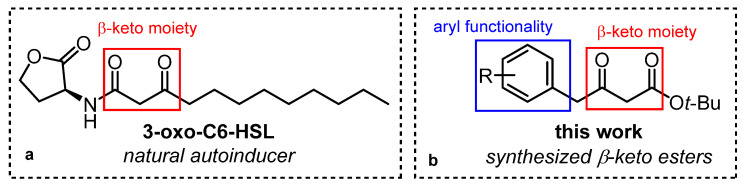
(**a**) Natural autoinducer employed by *V. fischeri*. (**b**) Our proposed QS inhibitor chemotype.

Figure 1 shows the chemical structure of the autoinducer (3-oxo-C6-HSL) discovered in the 1980s in the *Vibrio fischeri* lux genes of the QS system, which is responsible for bioluminescence [9]. Since many Gram-negative bacteria use *N*-acyl-homoserine lactones (AHLs) as autoinducers, these natural ligands are important starting points and inspiration for discovering new QS modulators. Numerous synthetic ligands have been designed, synthesised, and evaluated in this field, mimicking natural AHLs’ biological activity. The backbone of AHLs consists of three fundamental parts: a lactone ring, their amide function, and an acyl chain. It has been demonstrated that incorporating aryl functionality with electron-withdrawing groups in the acyl side chain converts many small molecules of AHL mimics into potent quorum-sensing inhibitors [10,11]. The central amide-connecting function of AHLs can be replaced with various nonnatural moieties, and these derivatives still retain their activity as synthetic LuxR-based quorum-sensing modulators [12,13]. On the other hand, the hydrolysis of the lactone present in AHLs by mammalian lactonases limits their potential as antivirulence drugs [14]. Several groups have identified nonnatural quorum-sensing modulators inspired by AHLs in which the native homoserine lactone group has been replaced by an aromatic group or carbocyclic rings [15,16].

Additionally, other QS systems important to several types of bacteria correspond to LuxS/AI-2 (S-ribosylhomocysteine lyase/autoinducer-2) and LasI-LasR with 3-oxo-C6-HSL as an autoinducer. The first system is implicated in biofilm formation in bacteria such as *Staphylococcus aureus* [17,18,19]. In contrast, the second is implicated in several characteristics related to bacteria’s pathogenicity, such as those of *Pseudomonas aeruginosa* and *S. aureus* [18,20,21].

Various commercial β-keto esters, the simplest structures with potential anti-QS activity, have also been analysed [22]. It has been observed that these compounds, incorporating an aryl substituent, interact with Lux-R-type proteins, thereby inhibiting quorum-sensing (QS) communication. Evaluating more β-keto esters will provide crucial information about the structure–activity relationships necessary for developing antivirulence agents. Therefore, this study designed and synthesised eight β-keto ester analogues of AHL natural autoinducers as potential quorum-sensing inhibitors (Figure 1). The compounds were chemically characterized, and a computational analysis of the reactivity and ADME (absorption, distribution, metabolism, and excretion) properties of the compounds as well as molecular docking and molecular dynamics calculations with the LasR and LuxS quorum-sensing (QS) proteins were carried out. An initial in vitro antimicrobial screening was carried out against human pathogenic and phytopathogenic bacteria.

## 2. Results and Discussion

### 2.1. Synthesis and Spectroscopic Characterisation of β-Keto Esters

**Scheme 1:**
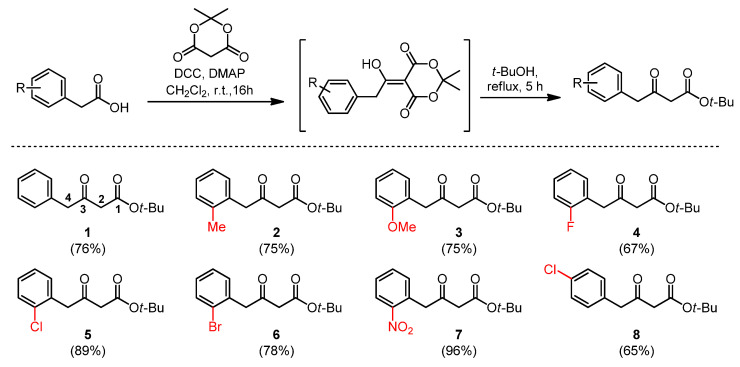
Synthesis of β-keto esters.

β-keto esters were synthesised from eight easily accessible commercial carboxylic acids, yielding between 65% and 96%. The choice of carboxylic acid was based on their substituents, with a primary focus on studying the impact of the *ortho* substitution of the phenyl group and its interaction with the active site of the LasR and LuxS QS proteins. We examined the interactions of the resonance-activating groups (**3**) or induction-activating groups (**2**, **5**, **6**, **7**, and **8**) and the deactivating groups (**4**) concerning this model compound (**1**). As shown in Scheme 1, target compounds **1**–**8** were synthesised. First of all, the commercially available phenylacetic acid derivatives were activated with 4-dimethylaminopyridine (DMAP) and *N*,*N*′-Dicyclohexylcarbodiimide (DCC) at 0 °C in dichloromethane solutions. Subsequently, condensation with Meldrum’s acid at room temperature overnight afforded the proper intermediates. Finally, the β-keto esters were efficiently obtained by refluxing the intermediates in *tert*-butanol.

To analyse these structures, we conducted NMR experiments using CDCl_3_ as the solvent. In all the compounds, we observed three singlet signals at 1.46–1.50 ppm, 3.37–3.55 ppm, and 3.82–4.25 ppm, which were assigned to the protons of the methyl group (–CH_3_) and the methylene groups (–CH_2_) of carbons 2 and 4, respectively. These signals confirmed that all the synthesised compounds existed in their keto form rather than their enolic form. However, the signals of the phenyl group varied depending on the type of substituent they had (see supporting information). Overall, all these analyses supported both the proposed structures and the purity of the β-keto esters, making these compounds suitable for bioassays.

### 2.2. Computational Analysis of the Reactivity and ADME Properties

It has been described that the quantitative relationship between the bioactivity/toxicity and the chemical structure of any compound can be established based on three aspects: [23] the compound’s hydrophobic, electronic, and steric characteristics. The weight of these three factors vary depending on the specific biological mechanism in which the compound is involved; particularly, the steric aspects can be relevant, for example, in specific interactions within the active site of the enzyme, which can be aborded through docking and molecular dynamics modelling [24]. Lipophilicity (hydrophobicity) has been the main focus in this area. However, electronic characteristics related to electrophile–nucleophile reactivity have also emerged as effective parameters to preliminarily assess the biological and toxicological activities of compounds of pharmacological interest [25,26,27].

#### 2.2.1. ADME Properties of the β-Keto Esters

We obtained the lipophilicity and other structural parameters, which allowed us to estimate the pharmacokinetic properties (ADME: absorption, distribution, metabolism, and excretion) of the compounds using the SwissADME server [28]. The descriptors predicted by the SwissADME server are presented in Table 1.

All the compounds presented acceptable parameters, meeting the drug-likeness criteria (Table 2) according to Lipinski’s rule of five [31,32]. It is worth noting that seven of the eight compounds should be able to permeate the blood–brain barrier, except for compound **7** (which contains a para-nitro group in the aromatic ring).

#### 2.2.2. Reactivity Indices Based on Electronic Structure

The description of the electronic aspect of the compounds is critical, for example, in aqueous toxicity mechanisms where nucleophile–electrophile interactions are the driving force [27]. It has been described that strong electrophiles can exert toxicity by covalently bonding with biological nucleophiles such as the cysteine or lysine amino-acid residues in enzymes, among others [37]. We focused mainly on the tendency of these compounds to react with potential biological nucleophiles by analysing their global electrophilicity and the local electrophilic sites within the compound.

**Figure 2:**
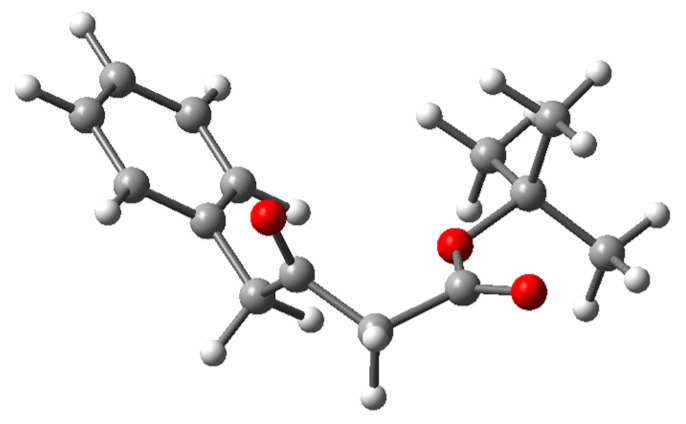
Molecular structure for the minimal energy conformation of compound **1**.

The previous experimental spectroscopic evidence shows that the compounds were in their keto form; therefore, we calculated the compounds considering only this tautomer. β-keto esters 1–8 were optimised at the DFT M062x/6-311+G(d,p) level. We conducted a conformational analysis on compound **1** to find the minimal energy conformation, identifying three stable conformations. The minimal energy conformation is presented in Figure 2.

To study the susceptibility of β-keto esters **1**–**8** to suffering a reaction with biological nucleophiles, we employed reactivity descriptors from conceptual DFT [27,38,39], which are presented in Table 3.

The results for the electronic chemical potential (*μ*) show that compound **3** presented the highest escaping tendency of its electrons from equilibrium, while compound **7** presents the lowest tendency. These results reflect the effect of the strongest electron-donating and electron-accepting substituents on the aromatic ring. The molecular hardness, a measure of the resistance of a compound to a charge transfer, shows that compound **4** presented the highest resistance, while compound **7** presented the lowest resistance to a charge transfer. This descriptor can be combined to produce a new descriptor, global electrophilicity (*ω*), which accounts for energy stabilisation due to the maximum electron flow from a donor environment. Compound **7** presented a remarkably high electrophilicity, indicating the highest tendency towards undergoing a nucleophilic attack from a potential biological nucleophile and, therefore, potential electrophilic toxicity [37]. Interestingly, there is also the comparison between isomers **5** and **8** (ortho- and para-Cl), where the last one presented the highest tendency towards undergoing a nucleophilic attack.

In order to analyse the local reactivity of the β-keto esters at more reactive positions, C-carbonyl atoms 1 and 3, we calculated the condensed Fukui functions for an electrophilic (*f_k_*^−^) and nucleophilic (*f_k_^+^*) attack and the condensed dual descriptor (*f_k_^2^*). We condensed the local hypersoftness ($s_{k}^{\left(2\right)}$) at these positions (Table 4). *f_k_*^−^ and *f_k_^+^* give us information about susceptibility to undergoing an electrophilic and nucleophilic attack separately, while the condensed dual descriptor (*f_k_^2^*) considers both reactivities simultaneously [38]. A positive value of *f_k_^2^* indicates an atom that tends to react with nucleophiles, while a negative value indicates an atom that tends to react with electrophiles. These descriptors, very useful for analysing the reactivity inside a molecule, do not allow a comparison among different molecules. To overcome this drawback, another local reactivity descriptor has been developed, local hypersoftness ($s_{k}^{\left(2\right)}$), which allows us to compare the local reactivity site among different molecules.

Experimentally, β-keto esters tend to react with nucleophiles at the esters’ C-carbonyl atom, for example, in the transesterification reaction [40]. The presence of *tert*-butyl can be a factor that alters this tendency through steric hindrance; however, it has been described that transesterification occurs without a problem in β-keto esters with a *tert*-butyl attached to their ester [40,41]. Our results for $s_{k}^{\left(2\right)}$ show that C1 was more susceptible to a nucleophilic attack in β-keto esters **1**, **2**, **3**, **4**, **5**, and **7**. On the other hand, for β-keto esters **6** and **8**, we found that their C3 was more electrophilic than their C1. These results suggest that compounds **6** and **8** could react differently than the rest of the series.

### 2.3. In Silico Analysis of Quorum-Sensing Activity

#### 2.3.1. Molecular Docking

To study how the compounds interact with the key targets involved in bacterial quorum sensing, we docked them in LasR and LuxS, and then we rescored the docking solutions using MM-GBSA. The results of these evaluations are presented in Table 5. This table details the docking score and binding-free-energy values for each of the eight compounds in their interaction with the proteins above. Lower binding-score values suggest a higher binding affinity between a compound and the corresponding protein. On the other hand, the MM-GBSA ΔGBind values provide a more accurate estimate of the binding free energy when considering the mechanistic and solvation terms. These values reflect the strength and stability of compound–protein interactions. Compounds with shallow docking scores and MM-GBSA ΔGBind values were identified in the analysis of the results, as is the case for compound **8**. These values highlight a high affinity and stability in the interaction of **8** with both proteins. β-Keto ester **8** shows a promising potential in efficiently interacting with these key proteins in bacterial communication.

#### 2.3.2. Molecular Dynamics

**Figure 3:**
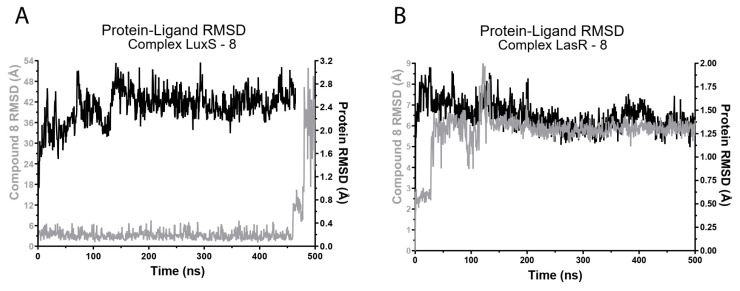
Root mean square deviation (RMSD) of the MD trajectories for compound **8**. (**A**) Complex of LuxS–**8** and (**B**) complex of LasR–**8**.

Molecular dynamics simulations were carried out to explore the interactions between compound **8** and both the LuxS and LasR proteins. Using simulations of a 500 ns duration for each system, the time evolution of the complexes was analysed in detail. In this context, the behaviour of compound **8** at the binding site of both proteins was examined, evaluating its stability and conformation throughout the simulations. The results provide fundamental information on the dynamics of these interactions and allow a more complete understanding of the interaction between compound **8** and the LuxS and LasR proteins. Specifically, remarkable stability was observed in the LuxS–compound **8** and LasR–compound **8** complexes over the 500 ns of the trajectories. This stability is evidenced by the root mean square deviation (RMSD) values of the protein backbones as shown in Figure 3A,B. The constancy in the structural conformation over this period suggests a robust interaction between compound **8** and both targets, reinforcing the validity of these interactions in the context of their long-lived molecular dynamics. In the case of LuxS, the RMSD of compound **8** remained consistently below 6 Å for the first 450 ns of the simulation. However, beyond this point, a significant increase in the RMSD of the ligand was observed, reaching approximately 40 Å. This change indicates an alteration in the interaction between the compound and LuxS. On the other hand, in the case of LasR, the RMSD of compound **8** remained relatively constant, in the range of 6 to 7 Å, throughout the entire 500 ns simulation. This points to a sustained and well-tuned ligand position at the LasR binding site, indicating a more persistent and stable interaction.

**Figure 4:**
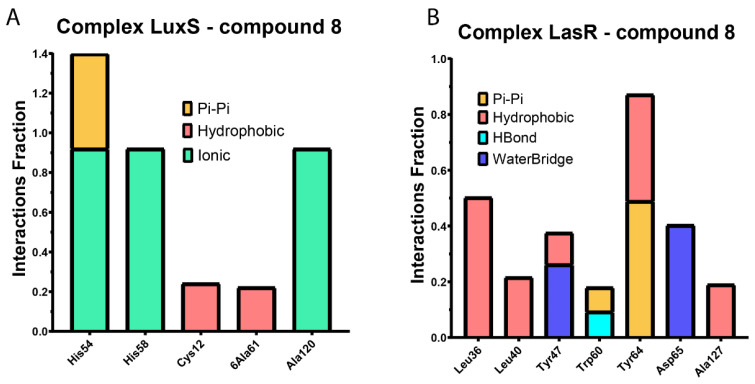
Interaction fractions over 500 ns of molecular dynamics simulations. Only residues that presented an interaction percentage higher than 20% over 500 ns of molecular dynamics simulations. (**A**) Complex of LuxS with **8** and (**B**) complex of LasR with **8**. The interactions of the protein with the ligand could be controlled throughout the simulation. Protein–ligand interactions are classified into four types: hydrogen bonds, hydrophobic interactions, ionic interactions, and water bridges. Each type of interaction contains more specific subtypes.

During the 500 ns MD, we analysed the interaction frequencies within the complexes. We focused mainly on the residues that exhibited interactions with a frequency higher than 20% during the trajectory. In the case of the LuxS–**8** complex (Figure 4A), different interactions were detected. These included π–π interactions involving His54; hydrophobic interactions with Ala60 and Ala120; and ionic interactions with His54, His58, and Cys126. In the LasR–**8** complex (Figure 4B), the residues Tyr64, Leu36, and Ala127 presented interactions with a significant frequency. In addition, π–π interactions were identified with Tyr64 and Trp60, whereas hydrogen-bonding interactions were established with Trp60. Notably, water-bridge interactions were observed with Tyr47 and Asp65. These interaction profiles contribute significantly to the stability of the LuxS–**8** and LasR–**8** complexes.

**Figure 5:**
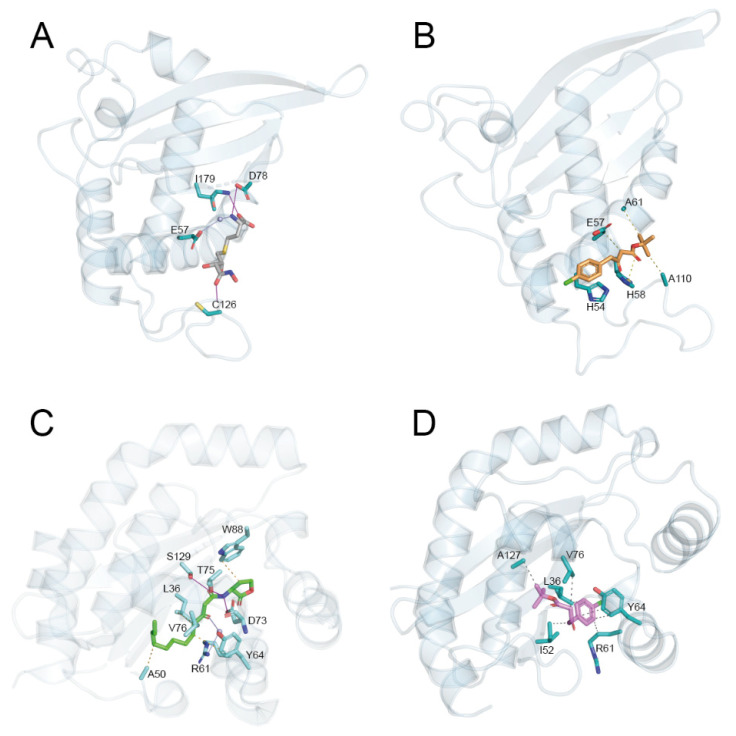
Representation of interactions. (**A**) LuxS–cocrystalised ligand complex (PDB code: 2FQO), (**B**) LuxS–**8** complex, (**C**) LasR–cocrystalised ligand complex (PDB code: 3IX3), and (**D**) LasR–**8** complex. Dashed black lines correspond to hydrophobic interactions; dashed yellow lines correspond to salt bridges; magenta lines correspond to hydrogen bonds; and grey lines correspond to water bridges.

Additionally, interaction modes corresponding to each complex are presented in Figure 5 where we also compare how the cocrystalised ligands interacted with the key proteins (Figure 5A,C). Compound **8** interacted with LuxS (Figure 5B) and LasR (Figure 5D) similar to the cocrystalised ligands; this suggests a possible shared affinity toward these residues regarding their ligand–protein interactions.

### 2.4. Antibacterial Activity

Following the theoretical assessment of the reactivity of the β-keto esters, an initial exploration was conducted to ascertain the antibacterial efficacy of these compounds against both pathogenic and phytopathogenic strains. Previous works have described various compounds’ antibacterial properties, potentially possessing antiquorum-sensing attributes [42,43]. Notably, several synthesised compounds could inhibit the activities of *P. aeruginosa*, *S. aureus*, *P. syringae*, and *A. tumefaciens*.

**Figure 6:**
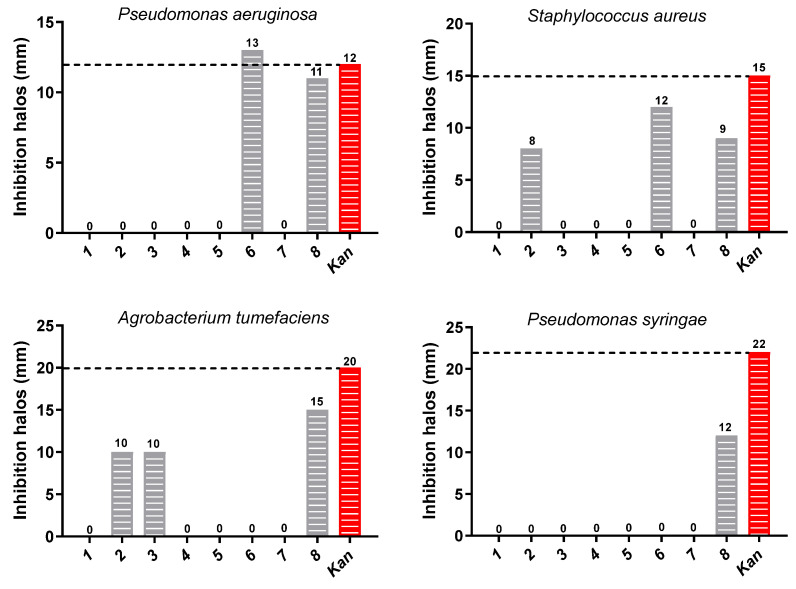
Inhibitory activity (mm) of β-keto ester compounds against pathogens and phytopathogenic bacteria. (■) β-keto ester compounds and (■) kanamycin, positive control.

Figure 6 illustrates the inhibitory diameters of the β-keto ester compounds against two common foodborne pathogens and two phytopathogenic bacteria. This technique is widely acknowledged as a useful semiquantitative method for assessing the sensitivity of microorganisms to specific compounds. Negative controls were employed using disks impregnated with acetone. To prevent any antimicrobial effects from acetone, these disks were dried under the flow of a biosafety chamber. Among the tested compounds, **2**, **3**, **6**, and **8** demonstrated inhibitory activity against these pathogens, with diameters ranging from 8 to 15 mm, classified as moderate/mild inhibitory activity compared to the values reported by other authors [44]. The minimum inhibitory concentration (MIC) and minimum bactericidal concentration (MBC) were determined only for the compounds that showed inhibition.

Table 6 reveals that the selected microorganisms exhibited susceptibility to the action of these compounds, with MIC values ranging from 0.08 mg/mL to 0.63 mg/mL and MBC values from 1.25 mg/mL to 5.00 mg/mL. Notably, β-keto ester **3** demonstrated higher resistance to antimicrobial activity against most of the strains, except for *A. tumefaciens*. Kanamycin (50 µg) served as the positive control for bacterial inhibition. Among the phytopathogenic bacteria, *P. syringae* (1.25 mg/mL MIC and 5.00 mg/mL MCB) and *A. tumefaciens* (0.08 mg/mL MIC and 1.25 mg/mL MCB) exhibited the highest susceptibility to compound **8**, requiring lower concentrations to inhibit bacterial growth. On the other hand, the bacteria of clinical importance, *S. aureus* (0.32 mg/mL MIC and 2.50 mg/mL MCB), were most susceptible to β-keto ester **8**.

## 3. Materials and Methods

### 3.1. Synthesis of β-Keto Esters

Synthesis of the β-keto esters used our group’s previously described method (see supporting information) [45].

***tert*-Butyl 3-oxo-4-(o-tolyl)butanoate (2).** This compound was prepared according to the general procedure described in the supporting information using 2-methylphenylacetic acid (0.30 g, 2.00 mmol), Meldrum’s acid (0.29 g, 2.00 mmol), DCC (0.45 g, 2.20 mmol), and DMAP (0.27 g, 2.20 mmol). Purification by column chromatography (0→1→2.5→5% EtOAc/hexane) gave β-keto ester **2** (0.37 g, 75%) as a yellow oil. ^1^H NMR (CDCl_3_, 400 MHz): δ 1.49 (s, 9H, CH_3_), 2.28 (s, 3H, CH_3_), 3.39 (s, 2H, H-2), 3.87 (s, 2H, H-4), and 7.15–7.22 (m, 4H, Ph). ^13^C NMR (CDCl_3_, 100 MHz): δ 19.7 (CH3), 28.0 (CH_3_), 48.3 (C-4), 49.7 (C-2), 82.1 (CCH_3_), 126.4, 127.7, 130.6, 132.4 (Ph), 166.4 (C-1), and 200.9 (C-3). HRMS calculated for C_15_H_20_O_3_ [M-H]^−^ was 247.1329; we found 247.1337.

***tert*-Butyl 4-(2-methoxyphenyl)-3-oxobutanoate (3).** This compound was prepared according to the general procedure described in the supporting information using 2-methoxyphenylacetic acid (0.30 g, 2.00 mmol), Meldrum’s acid (0.29 g, 2.00 mmol), DCC (0.45 g, 2.20 mmol), and DMAP (0.27 g, 2.20 mmol). Purification by column chromatography (0→2.5→5→10% EtOAc/hexane) gave β-keto ester **3** (0.37 g, 75%) as a yellow oil. ^1^H NMR (CDCl_3_, 400 MHz): δ 1.48 (s, 9H, CH_3_), 3.39 (s, 2H, H-2), 3.79 (s, 3H, CH_3_), 3.83 (s, 2H, H-4), 6.99–6.86 (q, 2H, Ph), 7.16 (d, *J* = 7.3 Hz, 1H, Ph), and 7.28 (t, *J* = 7.8 Hz, 1H, Ph). ^13^C NMR (CDCl_3_, 100 MHz): δ 28.1 (CH_3_), 44.8 (C-4), 49.8 (C-2), 55.5 (OCH_3_), 81.8 (CCH_3_), 110.7, 120.9, 128.9, 131.5 (Ph), 166.7 (C-1), and 201.5 (C-3). HRMS calculated for C_15_H_20_O_4_ [M-H]^−^ was 263.1278; we found 263.1295.

***tert*-Butyl 4-(2-Bromophenyl)-3-oxobutanoate (6).** This compound was prepared according to the general procedure described in the supporting information using 2-bromophenylacetic acid (0.59 g, 3.47 mmol), Meldrum’s acid (0.50 g, 3.47 mmol), DCC (0.79 g, 3.82 mmol), and DMAP (0.47 g, 3.82 mmol). Purification by column chromatography (0→1→2.5→5% EtOAc/hexane) gave β-keto ester **6** (1.09 g, 78%) as a yellow oil. ^1^H NMR (CDCl_3_, 400 MHz): δ 1.49 (s, 9H, CH_3_), 3.46 (s, 2H, H-2), 4.02 (s, 2H, H-4), 7.15-7.33 (m, 3H, Ph), and 7.60 (d, *J* = 8.0 Hz, 1H, Ph). ^13^C NMR (CDCl_3_, 100 MHz): δ 28.1 (CH_3_), 50.1 (C-4), 50.3 (C-2), 82.3 (C), 125.2, 127.8, 129.2, 132.0, 133.0, 134.1 (Ph), 166,4 (C-1), and 199.8 (C-3). HRMS calculated for C_14_H_17_BrO_3_ [M-H]^−^ was 311.0277; we found 311.0257.

### 3.2. ADME Properties’ Evaluation

The pharmacokinetic properties (ADME: absorption, distribution, metabolism, and excretion), physicochemical descriptors, and drug-likeness of the compounds were calculated using the SwissADME server [28]. Briefly, 42 descriptors were predicted for each compound, including physicochemical properties such as molecular weight, logP, solubility, and pharmacokinetic properties. Based on the descriptors obtained, the acceptability of the compounds based on bioavailability score (drug-likeness) could be assessed [28].

### 3.3. Quantum Chemical Calculation

*β*-keto esters were calculated using the Gaussian 09 [46] program package (Revision a.01; Gaussian, Inc.: Wallingford, CT, USA). No symmetry constraints were imposed on the optimisations performed at the DFT M06-2x/6-311+G(d,p) level. No imaginary vibrational frequencies were found at the optimised geometries, indicating true minima of the potential energy surfaces. Reactivity descriptors from Conceptual Density Functional Theory were obtained using the finite difference approximation (FDA) to analyse and compare the reactivity of *β*-keto esters.

Global reactivity descriptors were calculated as follows [27]:

(1) $$\mathrm{Electronic}\;\mathrm{Chemical}\;\mathrm{Potential}\;\mu =-0.5\;({IP}_{v}+{EA}_{v})$$

(2) $$\mathrm{Chemical}\;\mathrm{Hardness}\;\;\eta ={IP}_{v}-{EA}_{v}$$

(3) $$\mathrm{Electrophilicity}\;\;\;\;\omega =\frac{\mu^{2}}{\eta }$$

where *IP_v_* and *EA_v_* correspond to the vertical ionisation potential and vertical electron affinity, respectively.

Local reactivity descriptors were calculated as follows [38]:

(4) $$\mathrm{Fukui}\;\mathrm{Function}\;\mathrm{for}\;\mathrm{Nucleophilic}\;\mathrm{Attack}\;\;f_{k}^{+}=N_{k}\left(N\right)-N_{k}\left(N+1\right)$$

(5) $$\mathrm{Fukui}\;\mathrm{Function}\;\mathrm{for}\;\mathrm{Electrophilic}\;\mathrm{Attack}\;\;f_{k}^{-}=N_{k}\left(N-1\right)-N_{k}\left(N\right)$$

(6) $$\mathrm{Dual}\;\mathrm{Descript}\;\;\;\;\;f_{k}^{\left(2\right)}=f_{k}^{+}-f_{k}^{-}$$

(7) $$\mathrm{Local}\;\mathrm{Hypersoftness}\;\;\;\;s_{k}^{\left(2\right)}=\frac{f_{k}^{\left(2\right)}}{\eta^{2}}$$

where $N_{k}\left(N\right)$, $N_{k}\left(N+1\right)$, and $N_{k}\left(N-1\right)$ correspond to the electronic populations on atom k in neutral, radical anion, and radical cation species obtained through natural population analysis [39].

### 3.4. Docking and DM Calculations

To study the antibacterial potential of the β-keto esters, we selected the LasR and LuxS targets. These targets are involved in the pathogenicity of several bacteria, such as *P. aeruginosa* and *S. aureus* [14,18,20,21]. To determinate the binding site, we used the structure of LasR-OC1 cocrystalised with *N*-3-oxo-dodecanoyl-l-homoserine lactone (PDB code: 3IX3) [47] and LuxS cocrystalised with (*2S*)-2-amino-4-[(*2R*,*3R*)-2,3-dihydroxy-3-*N*-hydroxycarbamoyl-propylmercapto]butyric acid (PDB code: 2FQO) [48]. Prior to molecular docking calculations, proteins were prepared using the Protein Preparation Wizard tool included in Maestro. The ligands, waters (beyond 5 Å), and metals were removed from the structure; then, hydrogens were added, and ionisation states were calculated at pH 7.4 [49]. The proteins were energy-minimised with the OPLS4 force field. The centre of the grid boxes was located using the cocrystalised ligand in each structure. Molecular docking simulations were performed for LasR-OC1, with the outer edge of the grid set to 26 Å, and for LuxS, with the outer edge of the grid set to 22 Å. The standard precision function (SP) of Glide [50] was employed for docking simulations, and the best ten pose solutions per docked ligand were further subjected to postprocessing and rescoring by calculating binding free energy (ΔGbind) using the molecular-mechanics-generalised Born surface area (MM-GBSA) protocol in Prime [51]. The best complexes, according to ΔGbind, were subjected to 10 ns of equilibrium molecular dynamics (MD) simulations each using Desmond software [52] and the OPLS4 force field [53]. Then, 500 ns of production MD was performed for each complex. To prepare both systems, the complexes were solvated with pre-equilibrated single point charged (SPC) water molecules in a periodic-boundary-condition box. Neutralisation of the systems was done by adding Na^+^ or Cl^−^ counterions, and then, to simulate physiological conditions, a final concentration of 0.15 M NaCl was set. Each system was relaxed using the default Desmond relaxation protocol and then was equilibrated for 10 ns using the NPT ensemble at 1 atm and 300 K. A spring constant of 10.0 kcal × mol^−1^ × Å^−2^ was applied to the ligand and the protein. The last frame of equilibration MD was employed to perform production MD of 500 ns using the same conditions as those described above.

### 3.5. Antibacterial Activity

#### 3.5.1. Strain and Growth Conditions

β-keto esters were employed in assessing their antibacterial activity against several strains of bacteria, including the human pathogenic bacteria *Pseudomonas aeruginosa* (ATCC 19429) and *Staphylococcus aureus* (ATCC 29737) as well as the phytopathogenic bacteria *Pseudomonas syringae* (MF547632) and *Agrobacterium tumefaciens* (ATCC 19358). The bacteria were inoculated in nutrient broth containing 5.0 g/L of peptone and 3.0 g/L of meat extract followed by an incubation period of 18 h. Incubation temperatures were set to 25 °C for plant pathogens and to 35 °C for human pathogens. The incubation process was conducted with orbital shaking at 150 rpm utilising an incubator (MRC LOM-80).

#### 3.5.2. Paper-Disk Diffusion Method

The antibacterial properties of β-keto ester compounds were assessed following a method originally outlined by Parra et al. with modifications [34]. Initially, a stock solution of β-keto esters at a 20 mg/mL concentration was prepared using acetone as the solvent. Subsequently, 15 μL of this stock solution was applied to 5 mm sterile cellulose filter paper disks. In parallel, control disks impregnated with acetone were prepared to serve as negative controls. The impregnated disks were then dried within a biosafety chamber.

Disks containing 50 μg of kanamycin were employed as positive controls assessing bacterial inhibition. Fresh bacterial inoculum for each bacterial species was prepared as previously described, was diluted to a 0.5 McFarland standard (representing a bacterial concentration of 1.5 × 10^8^ CFU/mL), and was uniformly spread onto plates containing nutrient broth supplemented with 12 g/L of agar. The dried, impregnated disks were positioned equidistant from each other on the agar plates. Subsequently, the plates were incubated for 24 h at either 25 °C or 35 °C at the appropriate temperature. Following the incubation period, the diameter of bacterial-growth inhibition was measured, characterised by a transparent halo surrounding each disk where no bacterial growth was observed. To ensure precision and reproducibility, these tests were conducted in triplicate.

#### 3.5.3. Minimum Inhibitory Concentration (MIC)

The determination of the minimum concentration of β-keto esters required to inhibit bacterial growth followed the methodology detailed by Parra et al. [34]. The β-keto esters were evaluated using a concentration range from 0 to 10 mg/mL. Each concentration was prepared in a final working volume of 200 µL and was inoculated with the respective bacteria to be tested. These inoculated samples were then incubated in 96-well plates at either 25 °C or 35 °C at the appropriate temperature.

To serve as a control, nutrient broth without the compound was inoculated with each bacterium to monitor normal growth (growth control). Additionally, nutrient broth containing β-keto esters at concentrations ranging from 0 to 10 mg/mL, without bacterial inoculation, was employed to assess the compounds’ growth and sterility (negative control). To estimate the minimum inhibitory concentration (MIC) of acetone for each bacterium, an assay was conducted using acetone concentrations spanning from 0 to 90%. It was observed that acetone did not exhibit inhibitory effects on any of the four bacteria tested. After a 24 h incubation period, the lowest concentration of the compound at which no bacterial growth was detected was identified as the MIC for each bacterium.

#### 3.5.4. Minimum Bactericidal Concentration (MCB)

The bactericidal capacity of β-keto esters was assessed based on a method detailed by Parra et al. [34], focusing on the last three wells in the MIC assay that exhibited no bacterial growth. To determine the minimum concentration of β-keto esters where no growth was observed (MCB) for each microorganism, 100 µL of the bacterial cultures was plated on nutrient-broth plates supplemented with 15 g/L of agar. A culture that exhibited microbial growth in the MIC test was employed to serve as a growth control. Subsequently, the plates were incubated for 24 h at the appropriate temperature. Following incubation, the concentration of β-keto esters at which no growth was detected was recorded as the MCB for each microorganism.

## 4. Conclusions

We modelled and synthesised β-keto esters as antibacterial compounds in this work. The design was based on the structure of autoinducers of quorum-sensing Gram-negative bacteria. Eight β-keto ester analogues were synthesised with good yields, and they were spectroscopically characterised, showing that the compounds were only in their β-keto ester tautomer form. We carried out a computational analysis of the reactivity and ADME (absorption, distribution, metabolism, and excretion) properties of the compounds as well as molecular docking and molecular dynamics calculations with the LasR and LuxS quorum-sensing (QS) proteins, which are involved in bacterial resistance to antibiotics. The results show that all the compounds exhibited reliable ADME properties; none violated Lipinski’s rule. Based on the reactivity parameters obtained from the conceptual DFT calculations, only compound **7** could potentially present electrophile toxicity. The theoretical local reactivity study shows that compounds **6** and **8** reacted with nucleophiles at the keto C-carbonyl, unlike the rest of the series, which reacted with nucleophiles at the ester C-carbonyl. The molecular docking calculations show that compound **8** presented a better profile of affinity and stability in its interaction with the LasR and LuxS QS proteins. The molecular dynamics calculations allowed us to study the stability of the interaction between compound **8** and both proteins, being remarkable in both cases, particularly with LasR. An initial in vitro antimicrobial screening was performed against the human pathogenic bacteria *Pseudomonas aeruginosa* and *Staphylococcus aureus* as well as the phytopathogenic bacteria *Pseudomonas syringae* and *Agrobacterium tumefaciens*. Compounds 6 and 8 exhibited the most promising results in the in vitro antimicrobial screening against the panel of bacteria studied.

## Figures and Tables

**Table 1:** Physicochemical and pharmacokinetic descriptors calculated with SwissADME.

ID	Physicochemical Properties					Lipophilicity	Water Solubility	Pharmacokinetics		
	MW ^1^	Rot. Bond ^2^	HB-A ^3^	HB-D ^4^	TPSA ^5^	Consensus Log P_o/w_ ^6^	Solubility (mol/L)	GI Abs ^7^	BBB ^8^	log K_p_ (cm/s) ^9^
**1**	234.29	6	3	0	43.37	2.6	8.67 × 10^−5^	High	Yes	−5.92
**2**	248.32	6	3	0	43.37	2.97	3.56 × 10^−5^	High	Yes	−5.75
**3**	264.32	7	4	0	52.6	2.63	6.53 × 10^−5^	High	Yes	−6.12
**4**	252.28	6	4	0	43.37	2.96	4.58 × 10^−5^	High	Yes	−5.96
**5**	268.74	6	3	0	43.37	3.18	2.13 × 10^−5^	High	Yes	−5.68
**6**	313.19	6	3	0	43.37	3.24	1.29 × 10^−5^	High	Yes	−5.91
**7**	279.29	7	5	0	89.19	1.93	3.69 × 10^−4^	High	No	−6.31
**8**	268.74	6	3	0	43.37	3.19	2.13 × 10^−5^	High	Yes	−5.68

^1^ Molecular weight (g/mol); ^2^ number of rotatable bonds; ^3^ number of hydrogen-bond acceptors; ^4^ number of hydrogen-bond donors; ^5^ topological polar surface area [29]; ^6^ average of iLOGP, XLOGP, WLOGP, MLOGP, and SILICOS-IT predictions [28]; ^7^ gastrointestinal absorption; ^8^ blood–brain barrier permeation; and ^9^ skin permeation: QSPR model [30].

**Table 2:** Drug-likeness properties of the compounds calculated with SwissADME.

ID	Lipinski # Violations ^1^	Ghose # Violations ^2^	Veber # Violations ^3^	Egan # Violations ^4^	Muegge # Violations ^5^
**1**	0	0	0	0	0
**2**	0	0	0	0	0
**3**	0	0	0	0	0
**4**	0	0	0	0	0
**5**	0	0	0	0	0
**6**	0	0	0	0	0
**7**	0	0	0	0	0
**8**	0	0	0	0	0

**^1^** Lipinski (Pfizer) filter [32]: MW ≤ 500; MLOGP ≤ 4.15; N or O ≤ 10; and NH or OH ≤ 5. **^2^** Ghose filter [33]: 160 ≤ MW ≤ 480; −0.4 ≤ WLOGP ≤ 5.6; 40 ≤ MR ≤ 130; and 20 ≤ atoms ≤ 70. **^3^** Veber (GSK) filter [34]: rotatable bonds ≤ 10, and TPSA ≤ 140. **^4^** Egan (Pharmacia) filter [35]: WLOGP ≤ 5.88, and TPSA ≤ 131.6. **^5^** Muegge (Bayer) filter [36]: 200 ≤ MW ≤ 600; −2 ≤ XLOGP ≤ 5; TPSA ≤ 150; number of rings ≤ 7; number of carbon atoms > 4; number of heteroatoms > 1; and number of rotatable bonds ≤ 15.

**Table 3:** Vertical ionisation potential (*IPv*), vertical electron affinity (*EAv*), hardness (*η*), electronic chemical potential (*μ*), and global electrophilicity (*ω*) calculated at DFT M062x/6-311+G(d,p) level (all values in eV).

Compound	*IP_v_*	*EA_v_*	*η*	*μ*	*ω*
**1**	8.98	−0.77	9.75	−4.11	0.86
**2**	8.76	−0.79	9.55	−3.98	0.83
**3**	8.27	−0.89	9.17	−3.69	0.74
**4**	9.12	−0.76	9.88	−4.18	0.88
**5**	9.05	−0.73	9.78	−4.16	0.88
**6**	8.99	−0.72	9.72	−4.14	0.88
**7**	9.58	0.63	8.95	−5.10	1.46
**8**	8.91	−0.62	9.53	−4.15	0.90

**Table 4:** Condensed Fukui functions for an electrophilic (*f_k_*^−^) and nucleophilic (*f_k_^+^*) attack, condensed dual descriptor *f_k_^2^*, and condensed local hypersoftness $s_{k}^{\left(2\right)}$ over the C-carbonyl atoms of compounds **1**–**8** (all values in eV).

Compound	Atom	*f_k_^+^*	*f_k_* ^−^	*f_k_^2^*	$s_{k}^{\left(2\right)}$
**1**	C1	0.53	−0.18	0.71	5.56
	C3	0.84	0.21	0.63	4.90
**2**	C1	0.61	−0.17	0.78	6.33
	C3	0.25	0.16	0.09	0.77
**3**	C1	0.44	−0.16	0.60	5.25
	C3	−0.15	0.11	−0.25	−2.23
**4**	C1	0.34	−0.18	0.51	3.90
	C3	−0.11	0.25	−0.36	−2.71
**5**	C1	0.17	−0.16	0.33	2.54
	C3	0.16	0.16	0.00	0.03
**6**	C1	0.07	−0.17	0.24	1.86
	C3	0.35	0.09	0.26	2.02
**7**	C1	−0.14	−0.28	0.14	1.30
	C3	0.26	0.83	−0.57	−5.27
**8**	C1	0.33	−0.16	0.49	4.01
	C3	0.76	0.16	0.60	4.89

**Table 5:** Docking and binding free energy of compounds **1**–**8**.

Compound	LuxS		LasR	
	Docking Score (kcal/mol)	MMGBSA ΔG Bind (kcal/mol)	Docking Score (kcal/mol)	MMGBSA ΔG Bind (kcal/mol)
**1**	−3.781	−22.82	−6.405	−69.71
**2**	−3.475	−29.17	−2.742	−75.95
**3**	−4.052	−28.77	−5.253	−71.19
**4**	−4.265	−28.34	−7.291	−73.54
**5**	−3.981	−28.55	−7.439	−80.07
**6**	−4.011	−28.35	−4.335	−77.37
**7**	−1.188	−31.69	−3.555	−73.08
**8**	−4.085	−31.38	−4.649	−77.67

**Table 6:** Antibacterial activity of β-keto ester compounds.

Bacteria	MIC ^1^ (mg/mL)				Kan ^3^ (µg/mL)	MBC ^2^ (mg/mL)				Kan ^3^ (µg/mL)
	2	3	6	8		2	3	6	8	
**Pathogenic**										
*Pseudomonas aeruginosa* (ATCC 19429)	ND	ND	0.32	0.63	5.00	ND	ND	2.50	5.00	10.00
*Staphylococcus aureus* (ATCC 29737)	0.63	ND	0.63	0.32	2.50	5.00	ND	5.00	2.50	10.00
**Phytopathogenic**										
*Pseudomonas syringae* (MF547632)	ND	ND	ND	1.25	1.25	ND	ND	ND	5.00	2.50
*Agrobacterium tumefaciens* (ATCC 19358)	0.16	0.16	ND	0.08	1.25	2.50	2.50	ND	1.25	5.00

**^1^** Minimum inhibitory concentration. **^2^** Minimum bactericidal concentration. ND: inhibition not detected. ATCC: American Type Culture Collection (USA). MF547632 is the accession number to the Genbank of the respective bacteria. ^3^ Minimum bactericidal concentration (positive control).

## Data Availability

Data is contained within the article and Supplementary Material.

## Supplementary Materials

The following supporting information can be downloaded at: https://www.mdpi.com/article/10.3390/ph16101339/s1, Experimental and NMR data of compounds, optimized geometries of compounds, and Docking and Molecular Dynamics data of compounds.
