# Supplementary material for: Design of β-Keto Esters with Antibacterial Activity: Synthesis, In Vitro Evaluation, and Theoretical Assessment of Their Reactivity and Quorum-Sensing Inhibition Capacity
Source: Pharmaceuticals (Basel). 2023 Sep 22;16(10):1339. doi: 10.3390/ph16101339 (PMC10610512; doi:10.3390/ph16101339)
Supplement: Supplementary file 1 [file pharmaceuticals-16-01339-s001.zip › pharmaceuticals-2602068-supplementary.pdf]

Design of β-ketoesters with Antibacterial Activity: Synthesis, *In Vitro* Evaluation and Theoretical Assessment of Their Reactivity and Quorum Sensing Inhibition Capacity

Maximiliano Martínez-Cifuentes [1,*], Emmanuel Soto-Tapia [1], Camila Linares-Pipón [1], Ben Bradshaw [2], Paulina Valenzuela-Hormazabal [3], David Ramírez [3], Patricio Muñoz-Torres [4,*] and Claudio Parra [1,*]

[1] Departamento de Química Orgánica, Facultad de Ciencias Químicas, Universidad de Concepción, Ed-mundo Larenas 129, Concepción 4070371, Chile.
[2] Laboratori de Química Orgánica, Facultat de Farmàcia, IBUB, Universitat de Barcelona, Av. Joan XXIII, s/n, 08028 Barcelona, Spain
[3] Departamento de Farmacología, Facultad de Ciencias Biológicas, Universidad de Concepción, Concepción 4030000, Chile
[4] Laboratorio de Patología Vegetal y Bioproductos, Facultad de Ciencias Agronómicas, Universidad de Tarapacá, Av. General Velásquez 1775, 1000000 Arica, Chile

* Correspondence: maxmartinez@udec.cl; cparra@udec.cl; pmunozt@academicos.uta.cl

Supporting Information

Table of Contents

Experimental and NMR data of compounds **S2 – S6**
Optimized geometries of compounds **S7 – S13**
Docking and Molecular Dynamics data of compounds **S14 – S17**

Experimental

General. All reactions were carried out under an argon atmosphere with dry, freshly distilled solvents under anhyd conditions. Analytical thin-layer chromatography was performed on SiO_2 (Merck silica gel 60 F_{254}), and the spots were located with 1% aqueous KMnO4. Chromatography refers to flash chromatography was carried out on SiO_2 (SDS silica gel 60 ACC, 35-75 μm, 230-240 mesh ASTM). Drying of organic extracts during workup of reactions was performed over anhyd $MgSO_4$ except where stated otherwise. Evaporation of solvent was accomplished with a rotatory evaporator. NMR spectra were recorded in $CDCl_3$ on a Varian VNMRS 400. Chemical shifts of ^{1}H and ^{13}C NMR spectra are reported in ppm downfield (δ) from Me_4Si.

General Procedure: Synthesis of β-keto esters 1-8

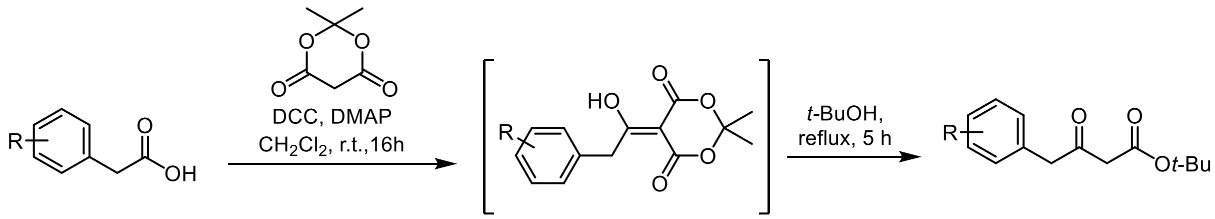

A solution of DCC (1.1 equiv) in CH_2Cl_2 (1 mL/mmol) was added slowly to a stirred solution of Meldrum's acid (1 equiv), the corresponding carboxylic acid compound (1 equiv), and DMAP (1.1 equiv) in CH_2Cl_2 (5 mL/mmol) at 0 °C. The reaction mixture was stirred at 0 °C for 16 h and the precipitated solid was removed by filtration and washed with CH_2Cl_2. The filtrate was washed with 1 M aq $NaHSO_4$, brine, dried (Na_2SO_4) and concentrated *in vacuo*. The residue was dissolved *tert*-butanol (4 mL/mmol) and the solution was refluxed under argon for 5 h. Concentration in vacuo and purification by column chromatography gave the corresponding β-keto ester product.

***tert*-Butyl 3-Oxo-4-phenylbutanoate (1).** This compound was prepared according to the above general procedure using DCC (1.00 g, 4.84 mmol), Meldrum's acid (0.64 g, 4.41 mmol), phenylacetic acid (0.60 g, 4.41 mmol), and DMAP (0.59 g, 4.84 mmol). Purification by column chromatography (0→2.5→5→10% EtOAc/hexane) gave β-keto ester **1** (0.78 g, 76%) as an amorphous solid. ^{1}H NMR (CDCl3, 400 MHz) δ 1.46 (s, 9H, CH3), 3.37 (s, 2H, H-2), 3.82 (s, 2H, H-4), 7.14-7.37 (m, 5H, o-ArH, p-ArH, m-ArH) ^{13}C NMR (CDCl3,100 MHz) δ 28.0 (CH3), 49.6 (C-4), 49.9 (C-2), 82.0 (C), 127.3 (o-Ar), 128.8 (m-Ar), 129.6 (p-Ar), 133.4 (ipso-Ar), 166.3 (C-1), 200.9 (C-3). Spectral data was identical to that previously reported [23].

***tert*-Butyl 4-(2-fluorophenyl)-3-oxobutanoate (4).** This compound was prepared according to the above general procedure using DCC (0.79 g, 3.82 mmol), Meldrum's acid (0.50 g, 3.47 mmol), 2-fluorophenylacetic acid (0.59 g, 3.47 mmol), and DMAP (0.47 g, 3.82 mmol). Purification by column chromatography (0→1→2.5→5% EtOAc/hexane) gave β-keto ester **4** (1.09 g, 67%) as a yellow oil. ^{1}H NMR (CDCl3, 400 MHz) δ 1.49 (s, 9H, CH3), 3.45 (s, 2H, H-2), 3.89 (s, 2H, H-4), 7.11 (dd, 2H, Ph), 7.18-7.23 (m, 1H, Ph), 7.27-7.31 (m, 1H, Ph). ^{13}C NMR (CDCl3,100 MHz) δ 28.0 (CH3), 43.1 (C-4), 50.0 (C-2), 82.3 (CCH3), 115.6, 124.4, 129.4, 131.9, (Ph), 166,3 (C-1), 199.7 (C-3). HRMS calcd for $C_{14}H_{17}FO_3$ $[M-H]^-$ 251.1078, found 251.1092. Spectral data was identical to that previously reported

***tert*-Butyl 4-(2-Chlorophenyl)-3-oxobutanoate (5).** This compound was prepared according to the above general procedure using DCC (0.79 g, 3.82 mmol), Meldrum's

acid (0.50 g, 3.47 mmol), 2-chlorophenylacetic acid (0.59 g, 3.47 mmol), and DMAP (0.47 g, 3.82 mmol). Purification by column chromatography (0→1→2.5→5% EtOAc/hexane) gave β-keto ester **5** (830 mg, 89%) as a yellow oil. 1H NMR (400 MHz, COSY) δ 1.47 (s, 9H, CH3), 3.43 (s, 2H, H-2), 3.97 (s, 2H, H-4), 7.25 (m, 3H, ArH), 7.39 (dd, J = 7.5, 3.2 Hz, 1H, ArH-3′) 13C NMR (100 MHz, HSQC) δ 27.9 (CH3), 47.5 (C-4), 50.1 (C-2), 82.1 (C), 127.0 (Ar-5′), 128.9 (Ar-4′), 129.5 (Ar-3′), 131.8 (Ar-6′), 134.5 (Ar-1′), 137.5 (Ar-2′), 166.2 (C-1), 199.5 (C-3). Spectral data was identical to that previously reported [23].

***tert*-Butyl 4-(2-Nitrophenyl)-3-oxobutanoate (7).** This compound was prepared according to the above general procedure using DCC (0.88 g, 4.25 mmol), Meldrum's acid (0.56 g, 3.86 mmol), 2-nitrophenylacetic acid (0.70 g, 3.86 mmol), and DMAP (0.52 g, 4.25 mmol). Purification by column chromatography (0→5→10→25% EtOAc/hexane) gave β-keto ester **7** (1.03 g, 96%) as an amorphous solid. 1H NMR (400 MHz, COSY) δ 1.50 (s, 9H, CH3), 3.55 (s, 2H, H-2), 4.25 (s, 2H, H-4), 7.31 (dd, J = 8.0, 1.2 Hz, 1H, Ar-6′), 7.47 (td, J = 8.0, 1.6 Hz, 1H, ArH-4′), 7.60 (td, J = 7.6, 1.6 Hz, 1H, ArH-5′), 8.13 (dd, J = 8.4, 1.2 Hz, 1H, ArH-3′); 13C NMR (100 MHz, HSQC) δ 28.0 (CH3), 47.7 (C-4), 50.6 (C-2), 82.3 (C), 125.3 (Ar-3′), 128.6 (Ar-4′), 129.8 (Ar-5′), 133.7 (Ar-6′), 166.2 (C-1), 198.6 (C-3). Spectral data was identical to that previously reported [23].

***tert*-Butyl 4-(4-Chlorophenyl)-3-oxobutanoate (8).** This compound was prepared according to the above general procedure using DCC (0.79 g, 3.82 mmol), Meldrum's acid (0.50 g, 3.47 mmol), 4-chlorophenylacetic acid (0.59 g, 3.47 mmol), and DMAP (0.47 g, 3.82 mmol). Purification by column chromatography (0→1→2.5→5% EtOAc/hexane) gave β-keto ester **8** (0.31 g, 65%) as a yellow oil. ^{1}H NMR (CDCl3, 400 MHz) δ 1.48 (s, 9H, CH3), 3.40 (s, 2H, H-2), 3.82 (s, 2H, H-4), 7.15 (d, 2H, Ph), 7.32 (d, 2H, Ph). ^{13}C NMR (CDCl3,100 MHz) δ 28.1 (CH3), 49.1 (C-4), 49.9 (C-2), 82.4 (C), 129.0, 131.1, 131.9, 133.4 (Ph), 166,4 (C-1), 200.4 (C-3). HRMS calcd for $C_{14}H_{17}ClO_3$ [M-H]$^-$ 267.0782, found 267.0766. Spectral data was identical to that previously reported.

^{1}H NMR (400 MHz, $CDCl_3$)

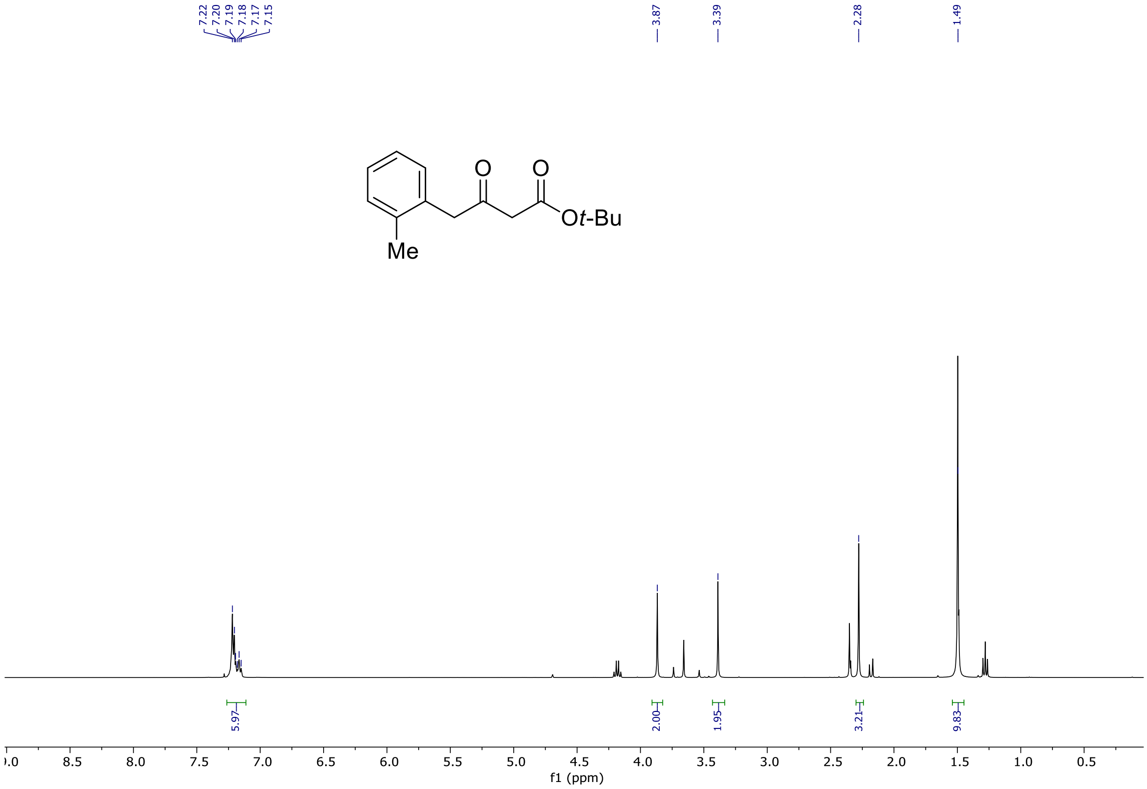

^{13}C NMR (100 MHz, $CDCl_3$)

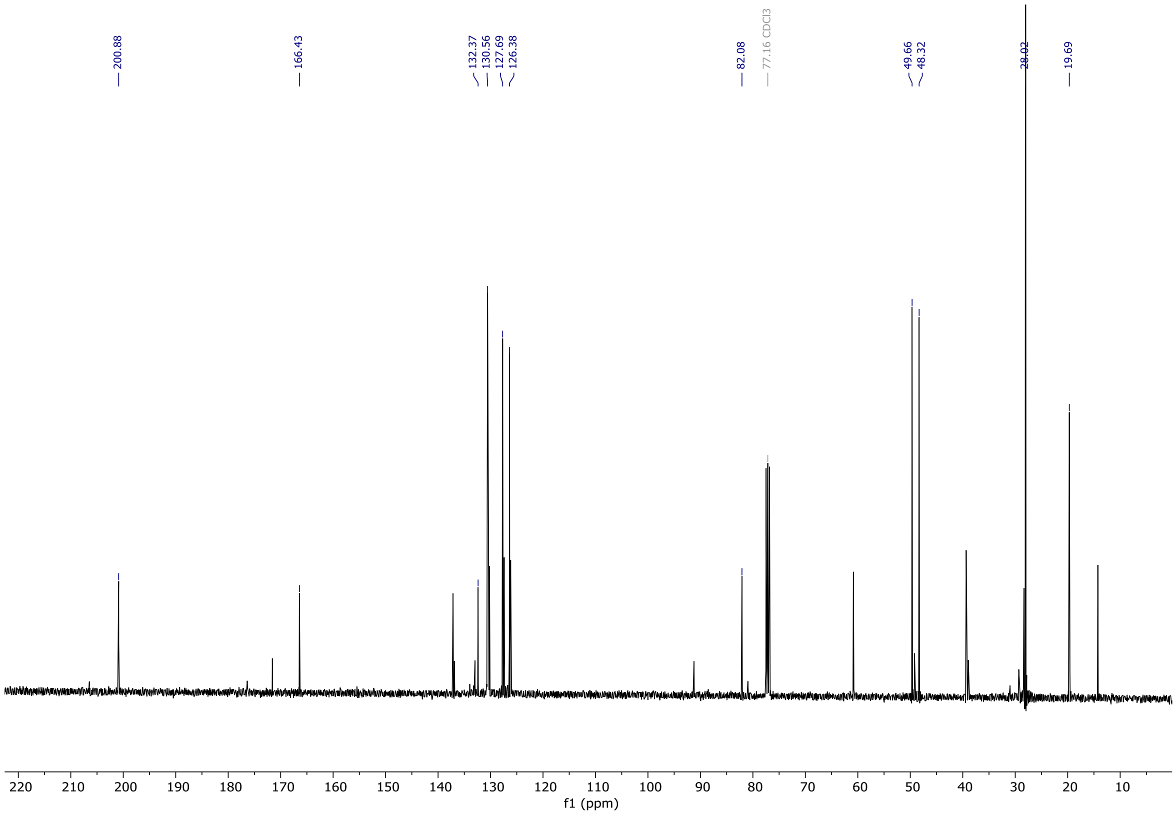

^{1}H NMR (400 MHz, CDCl$_3$)

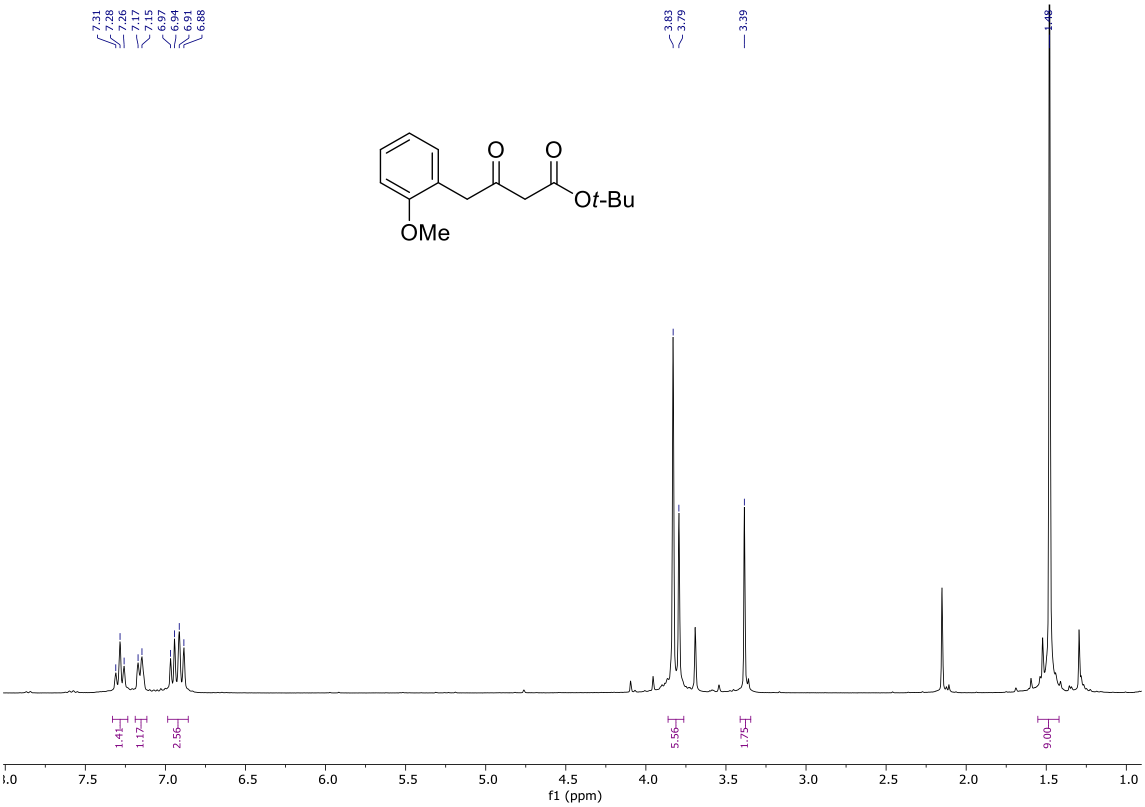

^{13}C NMR (100 MHz, CDCl$_3$)

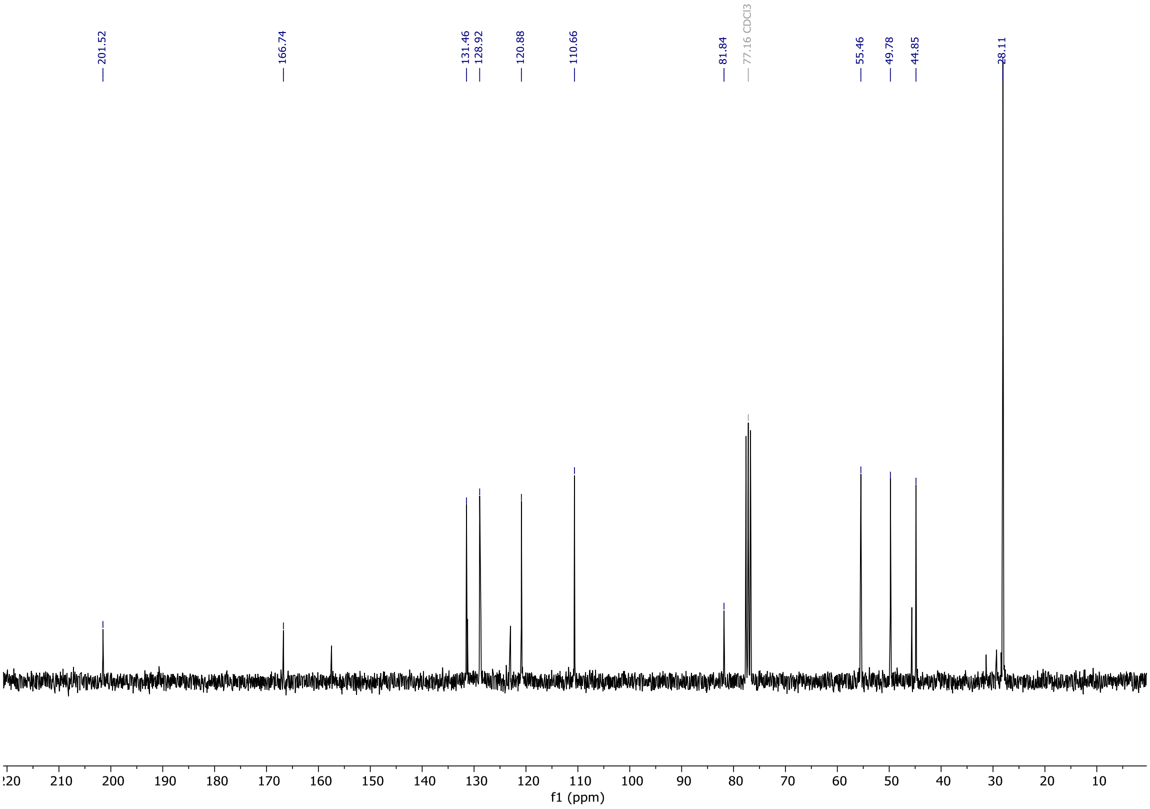

^{1}H NMR (400 MHz, CDCl$_3$)

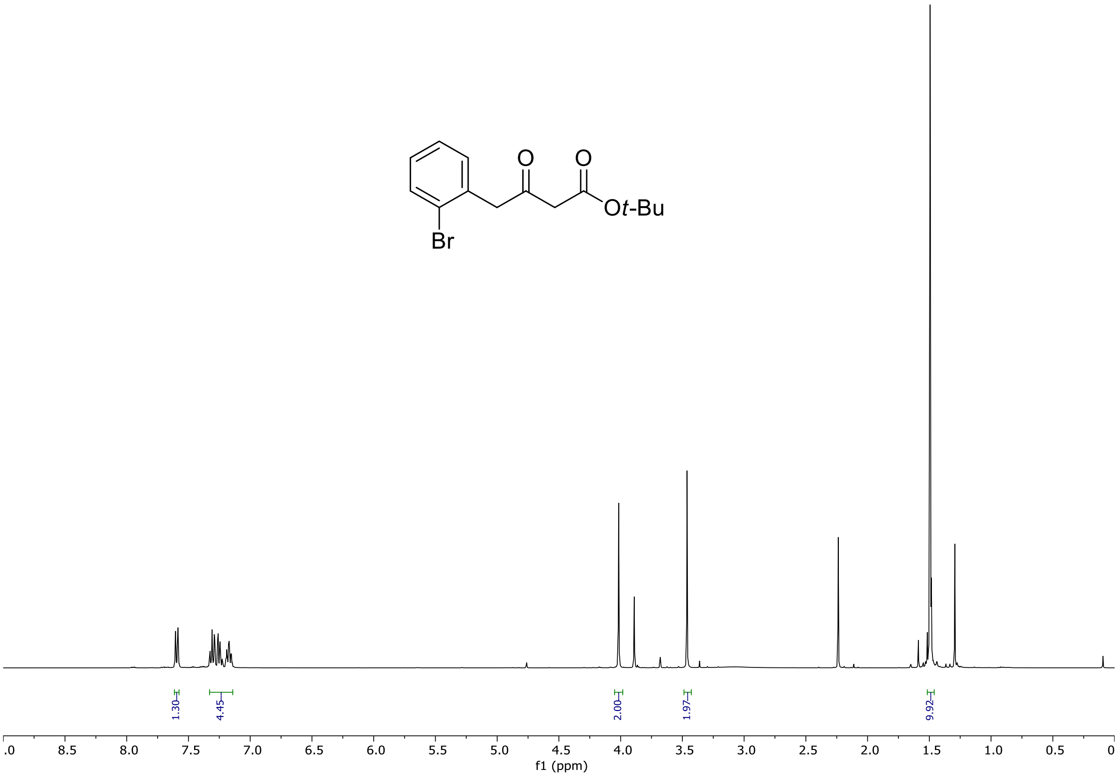

^{13}C NMR (100 MHz, CDCl$_3$)

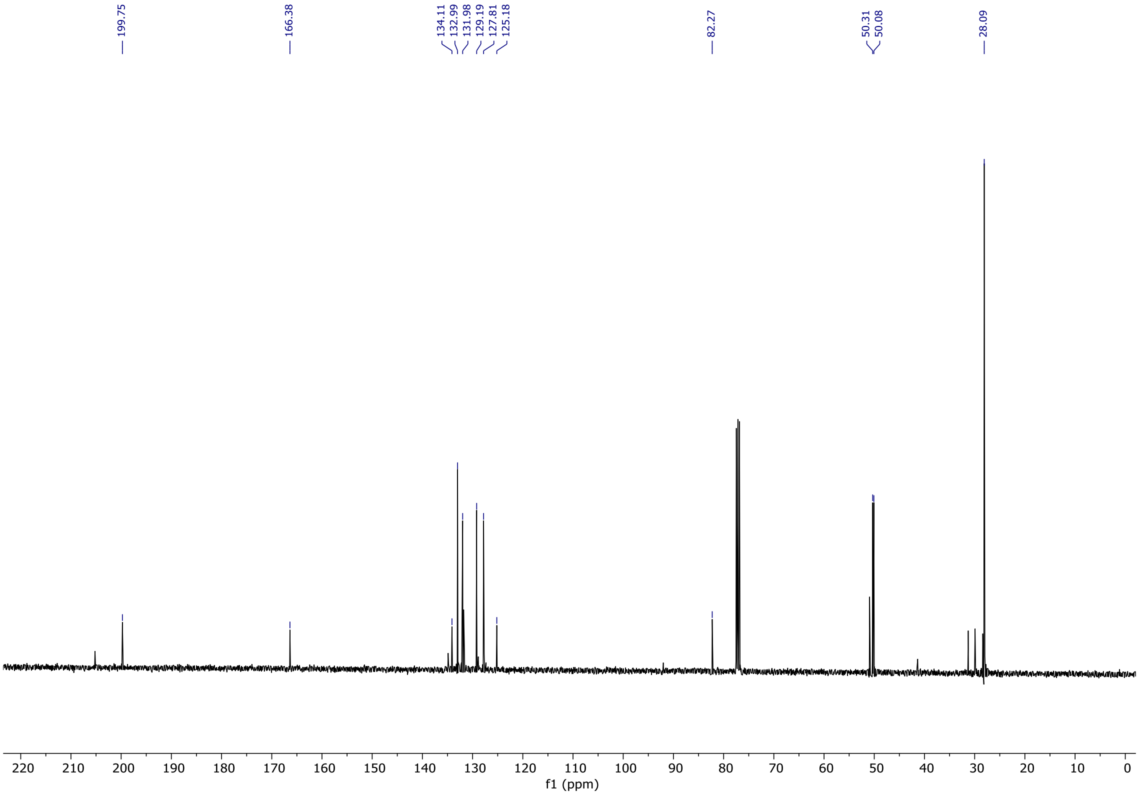

Optimized geometries at DFT M06-2x/6-311+G(d,p) level

Compound 1

C	-2.16063900	1.05955500	-0.77799400
C	-2.16411700	-0.31648900	-0.55714500
C	-3.20869800	-0.87918200	0.17795100
C	-4.22636900	-0.07833900	0.68139700
C	-4.21443800	1.29566300	0.45713800
C	-3.17903000	1.86385900	-0.27551600
H	-1.35329500	1.50481000	-1.34994900
H	-3.21524500	-1.94641900	0.36548200
H	-5.03048900	-0.52782400	1.25210800
H	-5.00932100	1.91863400	0.84956200
H	-3.16332000	2.93194700	-0.45849000
C	-1.04983000	-1.16777300	-1.10081200
H	-1.44773000	-1.97401600	-1.73296600
H	-0.38455000	-0.57619400	-1.73299300
C	-0.20825800	-1.86554600	-0.04032100
C	1.20397200	-2.26273000	-0.45723900
H	1.26420500	-2.42273900	-1.53776700
H	1.50431500	-3.17050500	0.06285100
C	2.20319300	-1.17560100	-0.09515400
O	-0.61094500	-2.12089300	1.06238700
O	3.32614400	-1.39445400	0.26220400
O	1.65301200	0.03460100	-0.24237500
C	2.37800700	1.25218600	0.13391800
C	2.72896500	1.20414300	1.61694300
H	3.48960000	0.45291700	1.82349800
H	3.10876300	2.18139700	1.92319600
H	1.83582600	0.98270300	2.20569200
C	3.60726600	1.42160500	-0.75130200
H	3.32049800	1.37928400	-1.80482700
H	4.05112800	2.40125900	-0.55919500
H	4.35243700	0.65376100	-0.55072600
C	1.35791000	2.34781500	-0.14181700
H	0.45441800	2.18418700	0.44924500
H	1.78053700	3.32028200	0.11803800
H	1.08891800	2.35747400	-1.20074900

Compound 2

C	-1.85809900	1.46829500	-0.46777400
C	-2.07311700	0.09272800	-0.51370600
C	-3.29826400	-0.43335200	-0.07688300
C	-4.27129600	0.44596800	0.39642200
C	-4.04894900	1.81800400	0.44454500
C	-2.83568900	2.33446800	0.00913300
H	-0.90841600	1.86312700	-0.81469800
H	-5.22041100	0.04545600	0.73703500

H	-4.82184100	2.47874200	0.81912500
H	-2.65064400	3.40160400	0.03730600
C	-0.97221300	-0.80141700	-1.01335300
H	-1.35325400	-1.57445600	-1.69402900
H	-0.23447700	-0.22950600	-1.58326200
C	-0.21533900	-1.53893500	0.08136100
C	1.09441500	-2.19925600	-0.33968800
H	1.10578200	-2.40122800	-1.41469800
H	1.23347000	-3.13118300	0.20538100
C	2.28436400	-1.30588900	-0.03102900
O	-0.59805900	-1.61836400	1.21775700
O	3.35234700	-1.71603200	0.32714100
O	1.97087700	-0.02165900	-0.23550100
C	2.93263300	1.05029600	0.03711200
C	3.33522500	1.02533900	1.50755400
H	3.94480200	0.15440000	1.74187700
H	3.90931800	1.92710300	1.73236400
H	2.44409900	1.02026300	2.13936500
C	4.13024000	0.92242400	-0.89728200
H	3.79557000	0.88013600	-1.93650800
H	4.76809000	1.80162100	-0.78065000
H	4.71621900	0.03313400	-0.67116700
C	2.13103700	2.30603900	-0.27621100
H	1.25942700	2.36974900	0.37834500
H	2.75186100	3.19054000	-0.12149500
H	1.79444300	2.29405400	-1.31553700
C	-3.56988000	-1.91474900	-0.11813700
H	-3.48400900	-2.30683900	-1.13600100
H	-2.86357600	-2.45719800	0.51405100
H	-4.57818200	-2.13087000	0.23564000

Compound 3

C	-1.63164900	1.85567700	-0.41629700
C	-1.81541500	0.48736400	-0.54259500
C	-3.04828600	-0.06698300	-0.16727100
C	-4.06552000	0.74388200	0.32702500
C	-3.85387100	2.11709800	0.44584300
C	-2.64337200	2.68012800	0.07433600
H	-0.67611500	2.28185200	-0.70498400
H	-5.01697400	0.32348100	0.62320100
H	-4.64989200	2.74240600	0.83238600
H	-2.48171300	3.74675700	0.16633100
C	-0.72524200	-0.41753800	-1.02910100
H	-1.10493500	-1.12316700	-1.77757500
H	0.07980900	0.15179300	-1.50264500
C	-0.09702700	-1.25784200	0.07279400
C	1.10893600	-2.09941400	-0.33759000
H	1.09252800	-2.31676800	-1.40953700
H	1.11247100	-3.03536700	0.21862400
C	2.41768300	-1.38715100	-0.04169700

O	-0.49228100	-1.27503300	1.20650000
O	3.42984400	-1.95021600	0.26910200
O	2.28039900	-0.06609500	-0.20142300
C	3.38716200	0.85628400	0.05831300
C	3.83872900	0.73519900	1.50949100
H	4.33633900	-0.21481700	1.69572400
H	4.53539400	1.54694800	1.73129200
H	2.97993800	0.82754300	2.17818200
C	4.52047500	0.59616100	-0.92745000
H	4.14597300	0.64152100	-1.95282500
H	5.28127700	1.37091600	-0.80738400
H	4.98073000	-0.37566500	-0.75552500
C	2.75009100	2.21639800	-0.19336100
H	1.92099500	2.37819400	0.49845200
H	3.48936000	3.00625100	-0.04726900
H	2.37331100	2.27595000	-1.21690800
O	-3.15769500	-1.41095600	-0.33879300
C	-4.31836200	-2.04684200	0.15408300
H	-4.17523800	-3.11158900	-0.01631700
H	-4.43898000	-1.86131500	1.22547600
H	-5.21240200	-1.71104300	-0.38093900

Compound 4

C	-1.98566200	1.50527600	-0.36185400
C	-2.06758200	0.12339100	-0.51203700
C	-3.26025000	-0.48558700	-0.14703300
C	-4.34636400	0.21305300	0.34312600
C	-4.23871700	1.59281900	0.48299000
C	-3.05957300	2.24005200	0.13016000
H	-1.06313100	2.00590600	-0.63629700
H	-5.24724200	-0.32522400	0.60873900
H	-5.07839900	2.15857000	0.86766600
H	-2.97571100	3.31435000	0.23817200
C	-0.91805700	-0.70364900	-1.00067800
H	-1.25064300	-1.44666400	-1.73544300
H	-0.16576400	-0.08080400	-1.49128900
C	-0.22599400	-1.47806700	0.11385200
C	1.07793600	-2.17336900	-0.26291300
H	1.09836700	-2.41419200	-1.33004800
H	1.18892000	-3.08873600	0.31549000
C	2.28532800	-1.29633000	0.02387300
O	-0.66015000	-1.55121800	1.23042100
O	3.34696000	-1.71849500	0.38632100
O	1.99724200	-0.01043300	-0.20906400
C	2.98873700	1.04704800	0.00402400
C	3.42871800	1.06516100	1.46366300
H	4.02820600	0.19133900	1.71243000
H	4.02447100	1.96331800	1.64211200
H	2.55474300	1.09836500	2.11815300
C	4.15858100	0.85840100	-0.95479300

H	3.79634400	0.79024200	-1.98332800
H	4.82137700	1.72394400	-0.88396100
H	4.72809600	-0.03782900	-0.71394000
C	2.20653800	2.30870200	-0.33526200
H	1.35750700	2.41920200	0.34249800
H	2.85102500	3.18396700	-0.23478300
H	1.83802500	2.26400000	-1.36272300
F	-3.36004800	-1.81999800	-0.29528700

Compound 5

C	-1.70010600	1.77578900	-0.34841500
C	-1.88993800	0.39922000	-0.46405700
C	-3.13731800	-0.11025300	-0.10470300
C	-4.16300100	0.70814200	0.34775100
C	-3.94625900	2.07632200	0.45336800
C	-2.71255800	2.61271900	0.10385900
H	-0.73511300	2.19012800	-0.62052600
H	-5.11615800	0.27031600	0.61534700
H	-4.74341400	2.71841000	0.80755100
H	-2.53840500	3.67871200	0.18339100
C	-0.77479100	-0.48923900	-0.92606000
H	-1.12903100	-1.22557900	-1.65618900
H	0.01037100	0.09311600	-1.41629700
C	-0.11511600	-1.26667100	0.20632800
C	1.10185300	-2.10113000	-0.17762800
H	1.06522200	-2.39036000	-1.23218800
H	1.13973500	-2.99882600	0.43746900
C	2.40220200	-1.34374700	0.03402200
O	-0.50423400	-1.23525100	1.34127800
O	3.43830300	-1.86915600	0.32893900
O	2.22652500	-0.03519500	-0.18171900
C	3.32620200	0.92012200	-0.02733600
C	3.84834500	0.88832000	1.40477200
H	4.36858400	-0.04322400	1.62042500
H	4.54332800	1.71921500	1.54582700
H	3.02204400	1.00920500	2.10878600
C	4.41684800	0.62605600	-1.05081900
H	3.99269200	0.59844900	-2.05734200
H	5.16046500	1.42550200	-1.01568200
H	4.91223000	-0.32109700	-0.84356200
C	2.65077000	2.25254800	-0.32246100
H	1.84954400	2.43554400	0.39643200
H	3.37861200	3.06278200	-0.24952400
H	2.23050900	2.25125100	-1.33083700
Cl	-3.44345700	-1.82622900	-0.23369400

Compound 6

C	-1.10045600	2.14345300	-0.29913700
C	-1.44208800	0.79548300	-0.41474300

C	-2.74264400	0.43181900	-0.06424900
C	-3.67310600	1.36376500	0.37671700
C	-3.30408800	2.69907500	0.48131700
C	-2.01428800	3.09106700	0.14284900
H	-0.09229400	2.44506300	-0.56308500
H	-4.67252500	1.04277100	0.63998100
H	-4.02672200	3.42729900	0.82920300
H	-1.72032500	4.13040000	0.22299200
C	-0.41873000	-0.20328400	-0.86373500
H	-0.84239800	-0.91894500	-1.57592100
H	0.41371100	0.29395000	-1.37073800
C	0.18564100	-1.00371600	0.28270100
C	1.27577300	-2.00028900	-0.09946000
H	1.19388300	-2.29223000	-1.15025600
H	1.20047300	-2.88601100	0.52900000
C	2.66017400	-1.40839600	0.10387100
O	-0.14012400	-0.86315200	1.42917600
O	3.60421400	-2.03125600	0.50024400
O	2.67035900	-0.11519400	-0.24213000
C	3.87704300	0.70354500	-0.10207700
C	4.31106800	0.74328400	1.35883100
H	4.69457600	-0.22059200	1.68874000
H	5.09717600	1.49296800	1.47464000
H	3.46813500	1.02918100	1.99211700
C	4.97262900	0.17603100	-1.02140400
H	4.60119800	0.09997300	-2.04603100
H	5.81143200	0.87578300	-1.01371900
H	5.32889100	-0.79939200	-0.69445600
C	3.40086600	2.07615400	-0.55821800
H	2.60101500	2.43350900	0.09362000
H	4.22688400	2.78866100	-0.52006800
H	3.02827900	2.02863900	-1.58390700
Br	-3.29467900	-1.38691900	-0.18257200

Compound 7

C	-1.45376500	1.93036700	-0.69721300
C	-1.67775900	0.55428600	-0.64519200
C	-2.86194000	0.14479000	-0.02740400
C	-3.77873700	1.03480800	0.51207900
C	-3.53441700	2.39748900	0.42363300
C	-2.36742700	2.84382300	-0.18405700
H	-0.53462500	2.28707400	-1.14868200
H	-4.66671800	0.64561300	0.99174500
H	-4.24904100	3.10176700	0.83054200
H	-2.16158200	3.90515100	-0.25378400
C	-0.62196300	-0.37725200	-1.17003300
H	-1.00782900	-1.02086300	-1.96482000
H	0.20780200	0.20074500	-1.58466800
C	-0.04475400	-1.29417700	-0.09944200
C	1.19044400	-2.09103200	-0.49574300

H	1.23216000	-2.23765700	-1.57872000
H	1.17650700	-3.05963900	0.00060600
C	2.46370700	-1.37345000	-0.07819400
O	-0.51032100	-1.38876200	1.00375100
O	3.45303800	-1.93078200	0.30328800
O	2.31910700	-0.04979600	-0.21793900
C	3.38094700	0.88219200	0.16943800
C	3.68048500	0.73490900	1.65690900
H	4.16798400	-0.21397600	1.87387200
H	4.34012000	1.54805100	1.96797600
H	2.75537600	0.80305100	2.23378200
C	4.61339800	0.65819100	-0.69868900
H	4.34437600	0.70905500	-1.75650700
H	5.34120100	1.44669600	-0.49387600
H	5.07465000	-0.30629100	-0.49231700
C	2.75058600	2.23756700	-0.12114400
H	1.84348100	2.36664600	0.47320900
H	3.45104900	3.03643500	0.12910900
H	2.49559900	2.31770400	-1.18051900
N	-3.21631300	-1.28759700	0.06800700
O	-3.91785000	-1.62777400	0.99075400
O	-2.81011600	-2.02931800	-0.80394100

Compound 8

C	1.59514400	-0.47360300	-1.00491700
C	1.37163100	0.87286900	-0.72740400
C	2.36295100	1.59412100	-0.06061400
C	3.55126800	0.98622900	0.32123300
C	3.74662200	-0.35843600	0.03281700
C	2.77971300	-1.09882800	-0.62984000
H	0.83329400	-1.04567700	-1.52328300
H	4.31990600	1.54346700	0.84140600
H	2.95353700	-2.14474600	-0.84830700
C	0.07638400	1.52267700	-1.12588400
H	0.26009700	2.41682300	-1.73773700
H	-0.52803300	0.84643900	-1.73373400
C	-0.77081200	2.00047200	0.04752800
C	-2.25928300	2.17856000	-0.22632700
H	-2.43823300	2.41173300	-1.28026300
H	-2.65893200	2.97742600	0.39547100
C	-3.03652700	0.91227100	0.09841700
O	-0.31463700	2.24992900	1.13046500
O	-4.15039400	0.91109000	0.54027200
O	-2.31111200	-0.17328900	-0.19207200
C	-2.81635300	-1.52240800	0.08357300
C	-3.07098000	-1.68300400	1.57801200
H	-3.91486300	-1.07882000	1.90697400
H	-3.28851700	-2.73258900	1.78842800
H	-2.18119300	-1.39734300	2.14355400
C	-4.06191200	-1.79266200	-0.75259900

H	-3.85707300	-1.59722300	-1.80789100
H	-4.33677300	-2.84480300	-0.64861800
H	-4.90040800	-1.17777700	-0.42970100
C	-1.66121800	-2.40789400	-0.36236000
H	-0.75736800	-2.16156200	0.19889600
H	-1.90933700	-3.45669000	-0.18898500
H	-1.46564300	-2.26850200	-1.42827600
H	2.19970500	2.63914000	0.17434200
Cl	5.23760000	-1.13019500	0.51285900

Docking and Molecular Dynamics

Energies of the docking score and MMGBSA ΔG bind

LuxS

Compound	Docking Score (Kcal/mol)	MMGBSA dG Bind (Kcal/mol)
1_1	-3.781	-22.82
1_2	-3.533	-22.15
1_3	-3.576	-21.5
1_4	-3.657	-20.8
1_5	-4.413	-20.43
1_6	-3.585	-20.39
1_7	-3.816	-18.17
1_8	-3.97	-17.89
1_9	-3.402	-17.45
2_1	-3.475	-29.17
2_2	-3.604	-26.23
2_3	-3.39	-24.63
2_4	-4.002	-24.32
2_5	-3.289	-20.67
2_6	-4.271	-19.04
2_7	-3.828	-18.76
2_8	-3.561	-16.56
3_1	-4.052	-28.77
3_2	-3.476	-25.3
3_3	-3.455	-24.57
3_4	-3.726	-23.94
3_5	-3.79	-23.24
3_6	-3.342	-23.22
3_7	-3.806	-22.41
3_8	-3.423	-22.27
3_9	-3.853	-21.67
3_10	-4.082	-20.46
4_1	-4.265	-28.34
4_2	-3.641	-25.72
4_3	-4.08	-24.98
4_4	-3.978	-24.27
4_5	-4.016	-24.12
4_6	-3.449	-21.76
4_7	-3.709	-20.8
4_8	-3.973	-20.19
4_9	-3.561	-19.74
4_10	-3.887	-18.57
5_1	-3.981	-28.55
5_2	-4.294	-28.13
5_3	-4.022	-27.83

5_4	-3.984	-27.48
5_5	-4.164	-27.46
5_6	-3.418	-26.94
5_7	-3.942	-26.6
5_8	-3.7	-25.86
5_9	-3.966	-24.56
5_10	-3.719	-23.28
6_1	-4.011	-28.35
6_2	-4.115	-27.8
6_3	-4.116	-27.56
6_4	-4.202	-27.5
6_5	-4.142	-26.65
6_6	-4.101	-26.04
6_7	-3.594	-25.13
6_8	-3.865	-24.76
6_9	-3.86	-24.16
6_10	-3.913	-24.13
7_1	-1.188	-31.69
7_2	-1.233	-29.91
7_3	-3.978	-28.41
7_4	-4.16	-27.88
7_5	-3.209	-24.03
7_6	-4.048	-23.78
7_7	-3.643	-23.73
7_8	-3.549	-23.32
7_9	-3.819	-23.14
7_10	-2.942	-18.71
8_1	-4.085	-31.38
8_2	-4.337	-29.43
8_3	-3.95	-27.79
8_4	-4.903	-27.42
8_5	-4.05	-27.1
8_6	-3.59	-26.14
8_7	-4.438	-25.84
8_8	-4.18	-25.78

LasR

Compound	Docking Score (Kcal/mol)	MMGBSA dG Bind (Kcal/mol)
1_1	-6.405	-69.71
1_2	-6.653	-69.52
1_3	-4.165	-67.92
1_4	-6.805	-67.11
1_5	-6.713	-65.36
1_6	-6.687	-63.55
1_7	-5.395	-63.18
1_8	-5.226	-61.78

1_9	-6.183	-61.22
1_10	-6.258	-60.87
2_1	-2.742	-75.95
2_2	-4.436	-73.99
2_3	-5.555	-72.78
2_4	-5.117	-72.42
2_5	-5.602	-65.91
2_6	-6.327	-65.08
2_7	-6.16	-64.11
2_8	-4.153	-63.56
2_9	-4.927	-63.05
3_1	-5.253	-71.19
3_2	-5.863	-68.06
3_3	-5.142	-67.05
3_4	-5.163	-66.96
3_5	-4.984	-66.68
3_6	-5.499	-66.58
3_7	-6.75	-66.11
3_8	-6.282	-65.42
3_9	-6.016	-60.31
3_10	-6.383	-58
4_1	-7.291	-73.54
4_2	-6.234	-73.5
4_3	-6.746	-71.67
4_4	-7.503	-69.71
4_5	-6.15	-67.06
4_6	-6.454	-66.98
4_7	-5.623	-66.34
4_8	-4.517	-65
4_9	-4.399	-63.55
4_10	-5.915	-58.34
5_1	-7.439	-80.07
5_2	-5.787	-75.84
5_3	-7.341	-74.39
5_4	-5.726	-72.56
5_5	-6.187	-72.22
5_6	-4.666	-72.14
5_7	-6.904	-68.54
5_8	-5.086	-68.5
5_9	-7.088	-68.23
5_10	-4.984	-62.93
6_1	-4.335	-77.37
6_2	-5.865	-76.88
6_3	-5.413	-76.79
6_4	-6.027	-74.84
6_5	-6	-74.6
6_6	-4.381	-71.78

6_7	-5.066	-70.14
6_8	-7.223	-68.99
6_9	-4.914	-64.57
7_1	-3.555	-73.08
7_2	-5.819	-69.85
7_3	-5.32	-69.85
7_4	-6.6	-68.05
7_5	-4.217	-67.86
7_6	-5.045	-67.07
7_7	-5.355	-66.02
7_8	-4.966	-62.39
7_9	-3.531	-58.69
7_10	-4.639	-57.75
8_1	-4.649	-77.67
8_2	-4.517	-77.22
8_3	-5.051	-73.77
8_4	-6.766	-72.77
8_5	-6.427	-72.67
8_6	-7.238	-72.63
8_7	-4.931	-70.49
8_8	-6.433	-70.37
8_9	-6.346	-66.48
